# Impact of Sex on Prediction of Asthma Attacks by Clinical Risk Factors and Type 2 Biomarkers

**DOI:** 10.1016/j.chest.2025.12.049

**Published:** 2026-01-31

**Authors:** Sebastian Riemann, Fleur L. Meulmeester, Samuel Mailhot-Larouche, Sanjay Ramakrishnan, Michael E. Wechsler, Jonathan Corren, Sarah E. Diver, Christopher E. Brightling, Mario Castro, Nicola A. Hanania, David J. Jackson, Neil Martin, Annette Laugerud, Deborah Clarke, Alison Moore, Megan E. Hardin, Cecile T.J. Holweg, Subhashini Allu, Timothy S.C. Hinks, Richard W. Beasley, Jacob K. Sont, Ewout W. Steyerberg, Ian D. Pavord, Guy Brusselle, Simon Couillard, The OxfoRd Asthma attaCk risk scaLE Meta-Analysis (ORACLE2) Consortium

**Affiliations:** a Department of Respiratory Medicine, Ghent University Hospital, Ghent, Belgium; b Department of Epidemiology, Eramus MC, Rotterdam, The Netherlands; c Department of Biomedical Data Sciences, Leiden University Medical Centre, Leiden; d Faculté de médecine et des sciences de la santé, Université de Sherbrooke, Québec, QC, Canada; e Institute for Respiratory Health, University of Western Australia, Perth, WA, Australia; f National Jewish Health, Denver, CO; g David Geffen School of Medicine at University of California Los Angeles, Los Angeles, CA; h Department of Respiratory Sciences, Institute for Lung Health, NIHR Respiratory BRC, University of Leicester, Leicester, England; i Department of Pulmonary Critical Care & Sleep Medicine, University of Kansas, Kansas City, KS; j Baylor College of Medicine, Houston, TX; k Guy’s Severe Asthma Centre, Guy’s and St. Thomas’ Hospitals, London; l AstraZeneca, Cambridge, England; m GlaxoSmithKline, London, England; n Sanofi, Cambridge, MA; o formerly Genentech, San Francisco, CA; p Novartis Healthcare Private Limited, Hyderabad, India; q Respiratory Medicine Unit and Oxford Respiratory NIHR BRC, Nuffield Department of Medicine, University of Oxford, Oxford, England; r Medical Research Institute of New Zealand, Wellington, New Zealand; s Julius Center, University Medical Center Utrecht, The Netherlands

**Keywords:** asthma, biomarkers, blood eosinophils, exacerbation, Feno, meta-analysis

## Abstract

**Background:**

Multiple clinical and inflammatory risk factors for asthma attacks have been identified, including attack history, comorbidities, blood eosinophil count (BEC), and exhaled nitric oxide (Feno). However, the impact of sex on their prognostic value is unclear.

**Research Question:**

Does sex modify prognostic values of clinical characteristics, BEC, and Feno for severe asthma attacks?

**Study Design and Methods:**

We conducted a patient-level meta-analysis of the control arms of 22 randomized asthma trials (Oxford Asthma Attack Risk Scale Meta-Analysis [ORACLE2], N = 6,510). Annualized severe asthma attack rates and (adjusted) rate ratios (aRRs) (95% CI) were estimated with sex-specific multivariable negative binomial models, adjusting for baseline demographics, clinical characteristics, and type 2 biomarkers (BEC and Feno). Interaction tests evaluated the influence of sex on other prognostic factors.

**Results:**

Among 4,140 female and 2,370 male patients, crude attack rates were higher in female patients than in male patients (0.90 vs 0.74 attacks/patient-year). Prior asthma attacks were the strongest predictor of future attacks in both sexes but with a higher aRR in male individuals (aRR [95%CI], 2.76 [1.97-3.88]) than in female individuals (1.66 [1.33-2.06]; interaction *P* = .03). In contrast, the prognostic utility of treatment intensity, BMI, lung function, smoking status, comorbidities, and type 2 biomarkers was similar in male and female individuals. The highest risk was seen in the combined high-BEC/high- Feno groups for both sexes.

**Interpretation:**

To our knowledge, this study is the first patient-level meta-analysis of prospectively collected clinical trial data to evaluate sex as a modifier of prognostic factors for asthma attacks. The overall annualized asthma attack rate was higher in female than male individuals. Prior attack history had stronger prognostic value for future attacks in male individuals, whereas other clinical risk factors and type 2 biomarkers (blood eosinophils and Feno) showed no major sex differences. These findings highlight that female individuals without prior attacks may face increased risk, and they have important implications for clinical research and practice.

**Take-Home Points**

**Research Question:** Does sex modify the prognostic value of clinical factors, blood eosinophils, and exhaled nitric oxide (Feno) for predicting severe asthma attacks?

**Results:** Female individuals had a higher annualized severe asthma attack rate than male individuals, with prior attack history being a stronger predictor in male individuals, whereas other clinical risk factors and type 2 biomarkers showed similar prognostic value across both sexes.

**Interpretation:** Our results show that female individuals without an asthma attack in the last year remain at elevated risk for future attacks, but biomarker-guided risk stratification using blood eosinophils and Feno can be applied similarly in both sexes, informing more equitable clinical decision-making and trial design.

Asthma is a heterogeneous chronic inflammatory airway disease affecting 260 million people worldwide, associated with significant burden.^1^ Notable differences occur between sexes across the lifespan.^2^ In childhood, asthma is more prevalent in boys, but this trend reverses around puberty, and by adulthood, approximately two-thirds of patients with asthma are female.^3^^,^^4^ This shift is thought to result from a complex interplay of hormonal influences, immune modulation, and differences in lung physiology.^5^, ^6^, ^7^ Despite these well-documented epidemiological patterns, the implications of sex differences for the risk and predictors of asthma attacks remain incompletely understood.

Among patients with asthma, several clinical characteristics and biomarkers have been identified as predictors of future asthma attacks, including prior attack history, disease severity, lung function impairment, and the type 2 (T2) inflammatory markers blood eosinophil count (BEC) and fractional exhaled nitric oxide (Feno).^8^, ^9^, ^10^, ^11^, ^12^ The 2025 update of the Global Initiative for Asthma (GINA), for the first time, recommends incorporating T2 biomarkers into the assessment of future attack risk. The shift from retrospective assessment (based on attack history or response to treatment) to prospective risk stratification using T2 biomarkers and clinical traits provides opportunities for an optimal management strategy in which interventions are proposed and implemented before permanent damage has occurred.^13^, ^14^, ^15^

Notably, female sex has been identified as an independent predictor of asthma attacks, even after adjusting for known risk factors.^8^^,^^16^ Conversely, T2-high adult-onset asthma is more frequently seen in male individuals.^17^ This raises the possibility that sex may not only influence disease prevalence and phenotype but also modify the prognostic value of established risk factors, including T2 biomarkers.^18^^,^^19^ Although T2 inflammation is a key driver of severe asthma and its associated attacks, potential sex-related differences in its impact on disease outcomes remain unexplored.^20^ Existing studies have either focused on single predictors or lacked interaction analyses to explore how sex may alter the relationship between clinical or inflammatory traits and attack risk.^19^

This study aims to investigate sex differences in prognostic values of clinical characteristics, and the T2 biomarkers BEC and Feno specifically, for severe attacks occurring in adults with asthma. Understanding such interactions is important to correctly weigh these parameters in risk-prediction models and to ensure that biomarker-guided management strategies are equally valid in male and female individuals.

## Study Design and Methods

### Study Design

We investigated the rate of severe asthma attacks (≥ 3 days systemic corticosteroids) and sex-specific prognostic effects in the control arms of randomized clinical trials (RCTs) in the Oxford Asthma Attack Risk Scale (ORACLE2) data set.

The ORACLE2 patient-level meta-analysis included 6,513 control arm participants aged ≥ 12 years from 22 RCTs (PROSPERO#:CRD42021245337, protocol published and initial analysis reported^21^). A complete overview of the included trials is provided in e-Table 1. These trials examined fixed treatment regimen effects on asthma attack rates over ≥ 6 months, with baseline BEC and Feno measurements. The control arm was the intervention with the lowest antiinflammatory therapy intensity after randomization (ie, randomized to no inhaled corticosteroids [ICSs], lowest dose ICSs, or ICSs plus placebo). Ethics approval was obtained from local trial committees. Anonymized data of ethically approved trials were used with additional review from the Oxford Tropical Research Ethics Committee who provided a waiver due to secondary analyses on anonymized data not requiring further ethical approval. This report follows the Reporting Recommendations for Tumor Marker Prognostic Studies (REMARK) checklist.^22^

### Data Analysis

Anonymized data were collected and imputed as previously described.^8^ Briefly, missing values were filled in using multiple imputation with the ‘mice’ package in R software across 10 data sets, pooling results with Rubin rules. Missing values for sex were excluded.

Baseline characteristics were described with median values (interquartile range [IQR]) by sex. Univariable and multivariable negative binomial models were fit to assess the relationships between characteristics and the annualized severe asthma attack rate (ASAAR) in male and female individuals separately. Multivariable models were adjusted for age, BMI, GINA treatment step, asthma attack history of the past year, FEV_1_% predicted before bronchodilation, Asthma Control Questionnaire-5 (ACQ-5), BEC, Feno, trial of enrollment as a factor, and log-transformed follow-up duration as offset variable. Results were displayed in forest plots. To examine the annualized attack rates across Feno and BEC (as continuous variables), spline curves were generated using restricted cubic splines (4 knots).

Time-to-first-attack analyses were performed using Kaplan–Meier survival curves and Cox proportional hazards models. Unadjusted Kaplan-Meier curves were generated by sex, and cumulative incidence at 1 year was reported. Multivariable Cox models were adjusted for age (using mean age as reference), ACQ-5 (mean ACQ-5 as reference), GINA treatment step (step 4 as reference), asthma attack history of the past year (no attacks as reference), FEV_1_% predicted (mean FEV_1_ as reference), BEC (mean BEC as reference), Feno (mean Feno as reference), and trial of enrollment (Clinical Study in Asthma Patients Receiving Triple Therapy in a Single Inhaler [CAPTAIN] trials as reference). Adjusted survival curves were derived from the Cox models.

In addition to sex-stratified multivariable models, an interaction analysis was performed in each of the 10 imputed data sets between sex and clinical characteristics as well as T2-biomarkers, after which results were pooled using Rubin’s rules. Multivariable models were adjusted for the same key characteristics previously indicated.

All statistical analyses were performed using R version 4.4.2, R code for data cleaning; analysis and visualization are provided on GitHub.^23^

## Results

Of the original ORACLE2 analytical data set (n = 6,513), we included 4,140 female and 2,370 male patients in the meta-analysis (3 patients were excluded because of missing data on sex). Female patients were slightly older (median, 50 [IQR, 40-59] vs 49 [37-59] years) and had a higher median BMI (28.2 [24.3-33.5] vs 27.6 [24.9-30.9]; Table 1). In particular, there were significantly more female individuals with BMI of 35-40 and ≥ 40 (obesity classes II and III).

**Table 1:** Descriptive Characteristics of Participants in ORACLE2

Variable	Female	Male	*P* Value
	N = 4,140 (64%)	N = 2,370 (36%)	
Age, y	**50 (40-59)**	**49 (37-59)**	**.013**
BMI	**28.2 (24.3-33.5)**	**27.6 (24.9-30.9)**	**.001**
BMI category			**< .001**
< 18.5	29/2,339 (1.2)	14/1,435 (1.0)	
18.5-24.9	641/2,339 (27)	359/1,435 (25)	
25-29.9	**719/2,239 (31)**	**613/1,435 (43)**	
30-34.9	496/2,339 (21)	306/1,435 (21)	
35-39.9	**287/2,339 (12)**	**107/1,435 (7)**	
≥ 40	**167/2,339 (7)**	**36/1,435 (2.5)**	
Smoking history			
Person who has never smoked	**3,456/4,065 (85)**	**1,739/2,328 (75)**	**< .001**
Person who formerly smoked	**593/4,065 (15)**	**567/2,328 (24)**	
Person who currently smokes	**16/4,065 (0.4)**	**22/2,328 (0.9)**	
Ethnicity			
Asian	299/3,030 (9.9)	203/1,751 (12)	
Black	**190/3,030 (6.3)**	**77/1,751 (4.4)**	**.104**
Other or multiple^a^	123/3,030 (4.1)	64/1,751 (3.7)	
White	2,418/3,030 (80)	1,407/1,751 (80)	
Baseline asthma medication			
Treatment step			
Step 1	**116/4,140 (2.8)**	**110/2,370 (4.6)**	**< .001**
Step 2	**171/4,140 (4.1)**	**144/2,370 (6.1)**	
Step 3	544/4,140 (13)	315/2,370 (13)	
Step 4	1615/4,140 (39)	939/2,370 (40%)	
Step 5	**1,694/4,140 (41)**	**862/2,370 (36)**	
mOCS use, n (%)	158/3,454 (4.6)	103/1,960 (5.3)	.33
Comorbidities			
Atopy history	1,667/2,628 (63)	871/1,377 (63)	.94
Allergy testing positive	**1,086/1,912 (57)**	**635/1,028 (62)**	**.01**
Eczema	219/2,087 (11)	111/1,094 (10)	.81
Allergic rhinitis	1,654/3,354 (49)	845/1,815 (47)	.06
Chronic rhinosinusitis	542/2,723 (20)	253/1,435 (18)	.08
Nasal polyposis	**363/2,728 (13)**	**228/1.430 (16)**	**.02**
Psychiatric disease	**328/2,165 (15)**	**92/1,132 (8.1)**	**< .001**
Asthma symptoms and history			
Asthma Control Questionnaire-5 score, median (IQR)	**2.6 (1.8-3.0)**	**2.4 (1.8-3.0)**	**< .001**
Severe attack in past 12 months	**3,389/4,140 (82)**	**1,821/2,370 (77)**	**< .001**
No. of severe attacks in past 12 months	**2 (1-2)**	**1 (0-1)**	**< .001**
Lung function			
Baseline FEV_1_% predicted	**64 (53-73)**	**62 (52-73)**	**< .001**
Baseline FEV_1_/FVC	**79 (78-81)**	**78 (76-80)**	**< .001**
FEV_1_ reversibility %	17 (10-28)	17 (9.9-26)	.07
Biomarkers			
Feno, median ppb	**22 (13-39)**	**26 (16-47)**	**< .001**
BEC, median cells/μL	**240 (140-410)**	**260 (150-430)**	**.004**
Total IgE, median ng/mL	**147 (54-382)**	**217 (84-539)**	**< .001**

Data are presented as n/N (%) or median (IQR) unless otherwise indicated. Between-group comparisons were performed using the Wilcoxon rank-sum test for continuous variables. Categorical variables were compared using the χ^2^ test. For categorical variables with more than 2 categories, post hoc analyses were performed using standardized residuals from the χ^2^ test. Statistically significant differnces (*P* < .05) are marked in bold. BEC = blood eosinophil count; Feno = fractional exhaled nitric oxide; IgE = immunoglobulin E; mOCS = maintenance oral corticosteroid.

a "Other" includes American Indian, Native Alaskan, Native Hawaiian, Maori and Pacific Islander.

To a limited extent, severe asthma (GINA step 5) was more prevalent in female individuals (41% vs 36%), as were comorbid allergic rhinitis (49% vs 47%) and depression or anxiety (15% vs 8%). Small differences were noted in baseline FEV_1_% predicted (62% in male participants vs 64% in female participants) and FEV_1_/FVC ratios (78% in male participants vs 79% in female participants). A higher proportion of male participants had smoked and quit (25% vs 15%).

Asthma control was consistently poorer in female individuals: the overall ACQ-5 score was higher (2.6 [1.8-3.0] vs 2.4 [1.8-3.0], *P* < .001), and female patients reported more symptoms across all individual components of the ACQ-5, including sleep awakenings, morning symptoms, activity limitation, dyspnea, and wheeze (all *P* ≤ .01; e-Table 2). A larger proportion of female individuals had experienced at least 1 severe asthma attack in the previous year (82% vs 77%; *P* < .001), with a higher median number of attacks (2 [1-2] vs 1 [0-1]; *P* < .001).

Feno levels at baseline were higher in male (26 [16-47] ppb) than female participants (22 [13-39] ppb). Similarly, blood eosinophil counts (BEC) were higher in male (260 [150-430] cells/μL) than in female participants (240 [140-410] cells/μL).

### Sex Differences in Prognostic Values of Clinical Risk Factors

**Figure 1:**
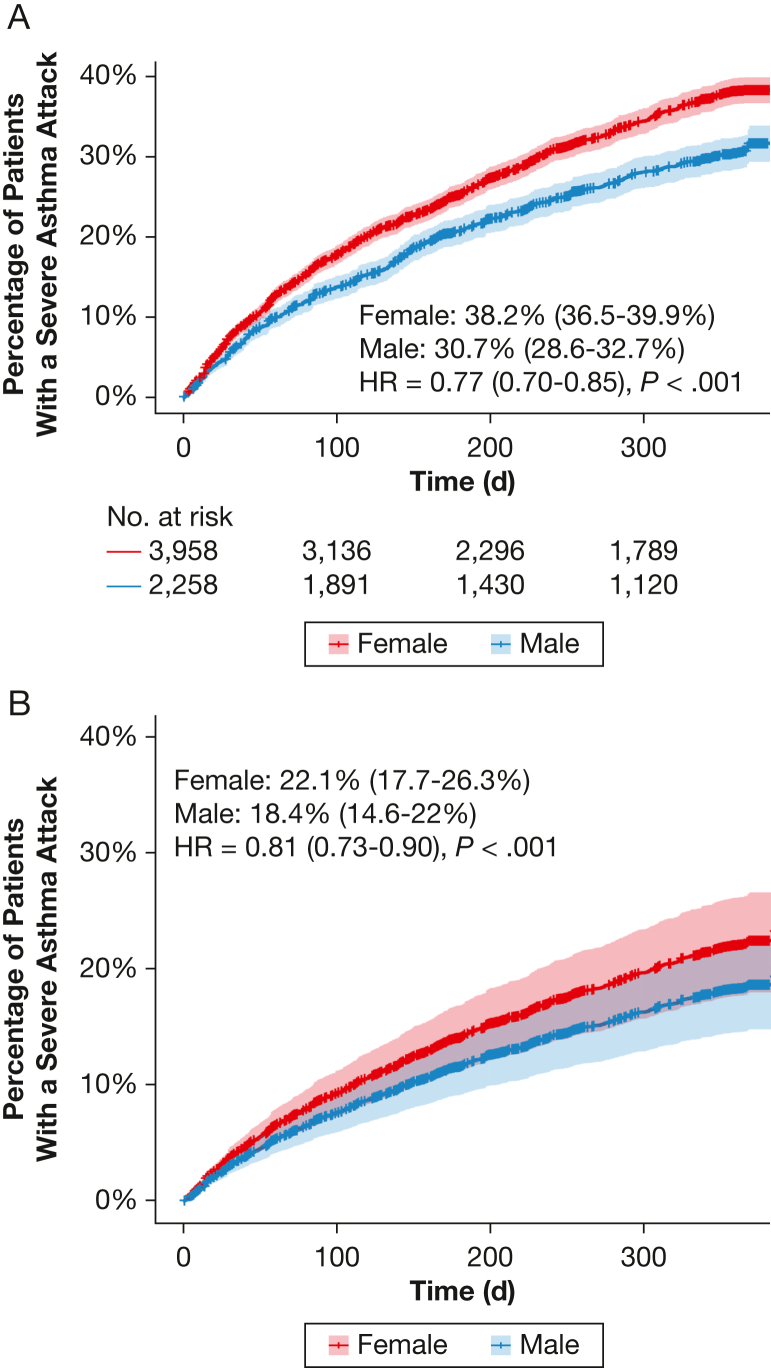
Proportion of male and female adults with ≥ 1 severe asthma attack in ORACLE2 over 1 year. Trials with < 12 months’ follow-up (Pediatric Asthma Controller Trial [PACT], Study of Efficacy and Safety of QAW039 in Patients With Severe Asthma Inadequately Controlled With Standard of Care Asthma Treatment [LUSTER-1, LUSTER-2]) were excluded. A, Unadjusted time-to-first attack. B, Adjusted for age, ACQ-5, FEV_1_ prebronchodilator, GINA step, Feno, and BEC. Shaded areas indicate 95% CIs. ACQ-5 = 5-item Asthma Control Questionnaire; BEC = blood eosinophil count; Feno = fractional exhaled nitric oxide; HR = hazard ratio.

A higher proportion of female individuals experienced at least 1 severe asthma attack (38% vs 31% at 1 year follow-up); both unadjusted (Fig 1A) and adjusted (Fig 1B) time to first attack were shorter in female individuals. The crude ASAAR was higher in female (0.90 attacks/patient-year) than in male participants (0.74 attacks/patient-year).

**Figure 2:**
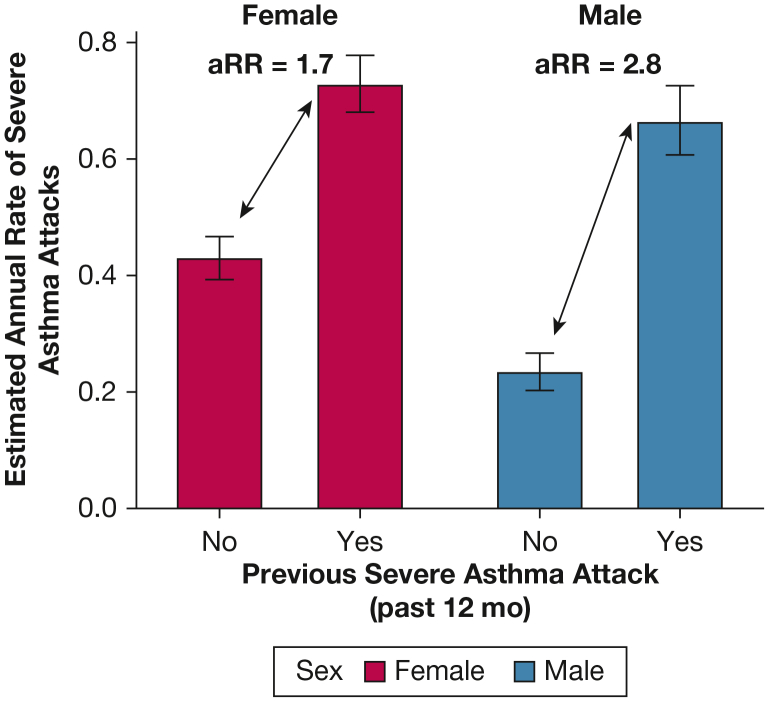
Sex-stratified adjusted annualised severe asthma attack rate (95% CI) by asthma attack history. Estimates use following reference values: mean age, mean BMI, mean baseline ACQ-5 score, mean FEV_1_ prebronchodilator, mean Feno, mean BEC, GINA treatment step 4, Clinical Study in Asthma Patients Receiving Triple Therapy in a Single Inhaler (CAPTAIN) trial, and duration of follow-up. ACQ-5 = 5-item Asthma Control Questionnaire; aRR = adjusted rate ratio; BEC = blood eosinophil count; Feno = fractional exhaled nitric oxide.

**Figure 3:**
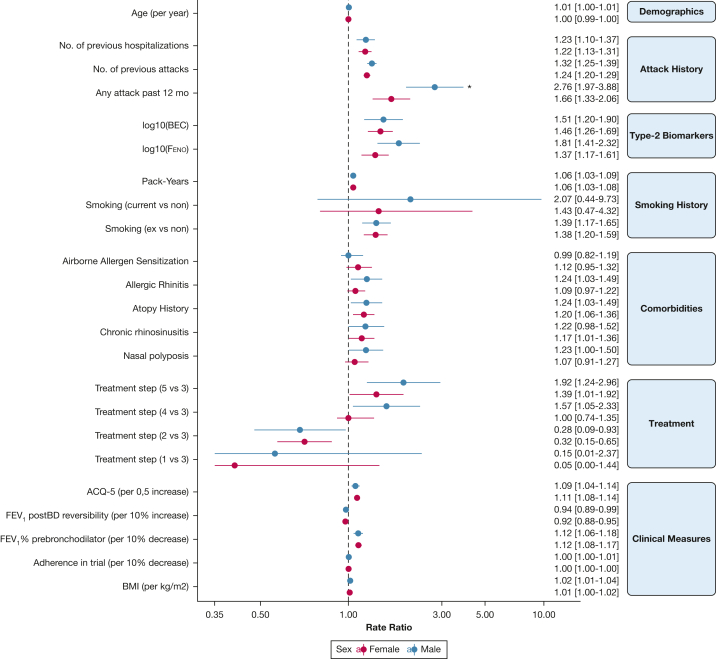
Adjusted rate ratios (aRRs; 95% CI) of annualized severe asthma attack rate according to different clinical variables. aRRs are derived from multivariable negative binomial models, adjusted for asthma attack in the past year (yes vs no), asthma severity (treatment steps 1-5), FEV_1_ prebronchodilator, ACQ-5 symptom score, BEC, Feno, adjusted enrolled trial as a factor, and follow-up duration as an offset variable. ACQ-5 = 5-item Asthma Control Questionnaire; BEC = blood eosinophil count; Feno = fractional exhaled nitric oxide, postBD = postbronchodilator. ∗*P* < .05 for sex interaction.

The adjusted rate ratio (aRR) for any asthma attack in the past 12 months was higher in male (2.8 [2.0-3.9]) than in female participants (1.7 [1.3-2.1]) (Fig 2). This was confirmed by a significant interaction between sex and attack history only (*P* = .03, e-Table 3). Figure 3 shows largely similar associations between male and female individuals for the other risk factors in multivariable analyses. Results from univariable analysis can be found in e-Figure 1. The respective aRR for the ASAAR for each unit increase in BMI were 1.02 [1.00-1.04] in male patients and 1.01 [1.00-1.02] in female patients (Fig 3). Relative to female patients with asthma with normal BMI, there was a higher risk of asthma attacks in female individuals with severe obesity (obesity class III, aRR 1.45 [1.09-1.91]). This rate ratio was similar for male individuals with severe obesity (1.40 [0.78-2.52]; e-Fig 2).

Although the associations between individual comorbidities and attack risk were comparable across sexes (e-Figs 3-6), and their baseline prevalence was also similar, the time to first severe asthma attack was shorter in female individuals than male individuals with the same number of comorbidities (e-Fig 7). Patients with 3 or more comorbidities at baseline experienced the highest risk, with over 40% experiencing at least 1 severe attack within 1 year.

### Prognostic Values of T2-Biomarkers by Sex

**Figure 4:**
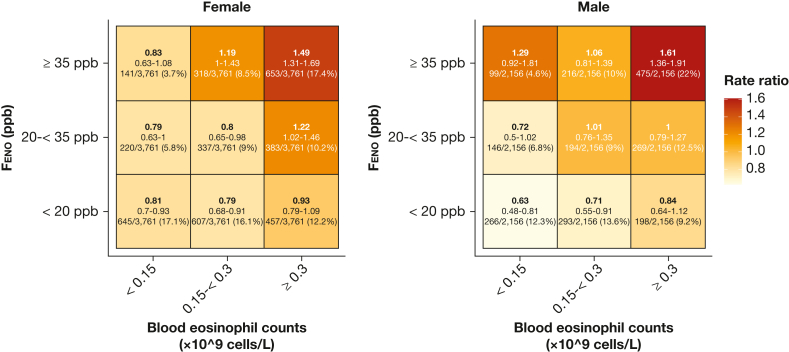
Sex-stratified adjusted annualized severe asthma attack rate (ASAAR [95% CI]) according to categories of BEC and Feno. Feno was stratified to low (< 20 ppb), intermediate (20-35 ppb), or high (≥ 35 ppb). ASAAR for each group is displayed in bold, followed by 95% CI and relative and absolute number of patients below. A negative binomial model was fitted for each imputed data set for the main predictors excluding Feno and BEC. Estimated risk was calculated using following reference values: mean age, mean BMI, mean baseline ACQ-5 score, mean FEV_1_% prebronchodilator, GINA treatment step 4, Clinical Study in Asthma Patients Receiving Triple Therapy in a Single Inhaler (CAPTAIN) trial and duration of follow-up. ACQ-5 = 5-item Asthma Control Questionnaire; BEC = blood eosinophil count; Feno = fractional exhaled nitric oxide.

In both sexes, adjusted ASAAR was higher for higher levels of the T2 biomarkers Feno and BEC. Among female individuals, those with Feno ≥ 35 ppb and BEC ≥ 0.3 × 10^9^ cells/L had the highest ASAAR (1.49 [1.31-1.69]), whereas those with low Feno (< 20 ppb) and low BEC (< 0.15 × 10^9^ cells/L) had the lowest ASAAR (0.81 [0.70-0.93]) (Fig 4). A similar pattern was observed in male individuals, with the highest ASAAR (1.61 [1.36-1.91]) also seen in the highest biomarker group and the lowest rate (0.63 [0.48-0.81]) in the lowest biomarker group. Analyses for Feno cutoffs at 25 ppb and 50 ppb yielded similar results (e-Fig 8). The synergistic effects of higher Feno and BEC on asthma attack rates were noted in both sexes (e-Figs 9-11).

A sensitivity analysis of biomarker distribution in the subset of trials that did not have a T2-biomarker target showed almost identical density plots for Feno and BEC in both sexes compared with the full data set (e-Fig 12, e-Table 4).

## Discussion

We investigated sex differences in the prognostic values of clinical characteristics and T2 biomarkers for the risk of (future) asthma attacks in the ORACLE2 individual patient-level data (IPD) meta-analysis, in which data were collected from the control arms of 22 RCTs. Overall, the annualized severe asthma attack rate was higher in female individuals than male individuals, and a history of asthma attacks in the past year more strongly predicted future asthma attacks in male compared to female individuals. Importantly, the statistical and prognostic significance of other clinical and inflammatory risk factors were largely similar across sexes.

A history of asthma attacks is 1 of the most consistently reported and robust predictors of future asthma attacks. Prior asthma attacks are a strong predictor of future attacks regardless of age or disease severity.^24^^,^^25^ However, these studies have generally not explored whether this prognostic relationship differs by sex, an important novel aspect addressed by our study. In our study, a history of asthma attacks appeared to be a stronger predictor of future attacks in male individuals, largely because female individuals without a recent attack (ie, within the prior 12 months) still had a relatively high rate of severe attacks during follow-up. From a clinical perspective, this difference highlights that even female individuals without asthma attacks in the prior year may face increased future risk, a factor clinicians might want to keep in mind. One possible explanation is that it is attributable to a higher baseline risk among female individuals without recent attack history, but it also may reflect the influence of more distant attacks. Prior research has shown that the risk associated with past attacks persists for several years, although it gradually diminishes over time.^25^ These findings suggest that more distant attack history, especially in female individuals, still may contribute meaningfully to future risk. This finding also calls for a reevaluation of how attack history is weighted in risk models.

Differences in levels of T2-biomarkers at baseline were consistent with previous reports, that is, slightly higher Feno and blood eosinophils in male individuals compared with female individuals.^26^, ^27^, ^28^ A similar increase in BEC or Feno, however, resulted in similar increases in the risk of asthma attacks in both sexes. This means that the prognostic utility of T2 biomarkers, whether assessed individually or synergistically, did not differ significantly by sex. In contrast, a recent retrospective real-world study reported a higher incidence of asthma attacks in female individuals with a T2-high status compared with male individuals with a T2-high status, whereas differences in attack rates among T2-low individuals were minimal.^19^ However, that study had some important limitations, including potential reporting bias because of the retrospective nature of the study; and the absence of Feno measurements, which limited their ability to reliably define T2-high status. Moreover, the analysis by Agarwal et al^19^ did not systematically assess interactions between sex and other clinical variables that could confound biomarker-outcome relationships. To our knowledge, our study is the first patient-level meta-analysis to investigate the interaction between sex, T2 biomarkers, and clinical characteristics in the context of asthma attacks, and it supports the notion that biomarker-based risk stratification can be applied in a similar way across sexes.

Previous studies indicated that adult female patients are more at risk for developing asthma and experiencing asthma attacks.^16^^,^^25^ To our knowledge, our novel findings confirm the increased risk of asthma attacks in female individuals, irrespective of the levels of T2 biomarkers: female individuals had both a higher annualized rate of asthma attacks and a shorter time to first attack compared with male individuals. In addition, female patients reported consistently poorer asthma control at baseline, with higher ACQ-5 scores and more symptoms across all individual domains as well as a higher frequency of severe attacks in the preceding year. Although female patients reported more symptoms, the mean differences in ACQ-5 scores were below the minimal clinically important difference (< 0.5). The relative associations between clinical risk factors (eg, obesity, comorbidities) and asthma attack risk were largely similar in both sexes. This suggests that the higher overall risk in female individuals is not attributable to stronger effects of individual risk factors, but rather to a higher baseline susceptibility. This might be at least partially attributable to the higher baseline prevalence of risk factors such as obesity and other comorbidities such as anxiety and depression in female individuals.^29^ The sex difference in time to first attack largely disappeared after adjusting for the core set of clinical measures, confirming the important contribution of baseline risk factors to the difference in risk profile between both sexes. This distinction has important clinical implications: although the intensity of clinical management should remain consistent across sexes, targeted screening for common comorbidities in female patients (eg, mood disorders, obesity) may yield greater preventive benefit. Regarding treatment, our data support the previous suggestions to primary care physicians to provide personalized asthma care for all patients regardless of sex.^30^

The higher risk of asthma attacks in female patients as compared with male individuals also could be the result of genetic, immunological, or hormonal influences not captured by the factors assessed in this meta-analysis. Female steroid hormones (ie, estrogen) in particular have been associated with enhanced T2-inflammation, potentially leading to a higher frequency of asthma attacks.^6^ Estrogen suppression (eg, use of contraceptives) has been linked to a decrease in asthma activity, whereas use of hormone replacement therapy in postmenopausal female individuals has been associated with increased asthma incidence and severity.^31^ Testosterone, however, has been linked to decreased (T2-) inflammation and a protective role against asthma in both male and female individuals.^32^^,^^33^ Further translational research into the sex differences of asthma is needed to elucidate the higher baseline risk of asthma attacks in female patients that we could not (fully) explain by differences in clinical risk factors or T2 biomarkers alone.

Obesity is a known risk factor for asthma attacks as a result of increased systemic inflammation, altered physiology, and an increase in co-morbidities, which further increase the risk of asthma attacks.^34^^,^^35^ Our findings show that the highest attack rate was in those with extreme obesity, but limited differences were observed between the attack risk of obesity in both sexes. However, because of the small number of participants with extreme obesity (BMI ≥ 40), the results should be interpreted with caution regarding this subgroup.

An interesting paradox also arises regarding BMI. Although we showed that the highest risk of asthma attacks and thus the highest unmet clinical need was present in patients with asthma and severe obesity (BMI ≥ 40), this group is sometimes excluded from clinical trials, partly because of concerns about pharmacokinetic variability or reduced treatment response. In the ORACLE2 analysis, this was the case for the Study of Lebrikizumab in Patients Whose Asthma is Uncontrolled With Inhaled Corticosteroids and A Second Controller Medication (LUTE, VERSE) and the Study to Evaluate the Efficacy and Safety of MEDI9929 (AMG 157) in Adult Subjects With Inadequately Controlled, Severe Asthma (PATHWAY).^36^^,^^37^ Because obesity rates are rising globally, clinical trials might consider either proactively including patients with severe obesity or designing parallel studies that specifically address the unique pathophysiology and treatment responses in this subgroup. Notably, prognostic effects of BMI were similar across both sexes in our control arm data set, nuancing previous observations on the role of obesity in asthma control. However, because no data from the active treatment arms were included, we cannot assess differential treatment responses. Future research should include intervention arms to evaluate whether female and male individuals with obesity respond differently to biomarker-guided therapeutic strategies.^38^^,^^39^

Finally, our results raise a few important considerations for the design and interpretation of clinical trials in (severe) asthma regarding both sex and obesity. Baseline imbalances in comorbidities (eg, higher prevalence of anxiety or depression in female individuals) may create “sex-driven high-risk” groups. Given that many asthma trials tend to recruit mostly female participants, this disparity might further contribute to an imbalanced attack risk. In future clinical trials, it may be worthwhile to aim for more balanced recruitment by sex, or at least to employ sex-stratified randomization.^29^ In this way, increased attack risks in the control arms would reflect disease-specific variables (eg, T2 inflammation).

The strengths and limitations of the ORACLE2 IPD meta-analysis have been discussed previously.^8^ In short, the collaboration between academic, public, and pharmaceutical partners provides a platform in which high-quality prospective IPD from RCTs is used. Conversely, only patients from the control arms of the (included) RCTs were analyzed. This could have potentially introduced placebo bias, typically seen in RCTs. We performed a sensitivity analysis of T2-biomarker levels in the trials that did not have an inclusion criterium or biomarker target for T2-biomarkers and found almost identical levels of both Feno and BEC in the subgroups compared with the complete data set. This supports the generalizability of our results irrespective of biomarker levels. One important caveat in the current study was the absence of information on gender: we only analyzed the prognostic effects of sex at birth.

## Interpretation

The annualized asthma attack rate was higher in female than male patients. We found a stronger prognostic value for future asthma attacks of prior attack history in male individuals, whereas there were no major sex differences in the prognostic value of other clinical risk factors and T2 biomarkers (blood eosinophils and Feno). These findings highlight that female patients without asthma attacks in the past year may face increased future risk and have important implications for both clinical research and clinical practice.

## Funding/Support

Supported by the 10.13039/501100000272National Institute for Health Research (NIHR), Oxford Biomedical Research Centre, Association Pulmonaire du Québec (APQ), the Fonds de Recherche du Québec—Santé (FRQS), the Québec Air-Intersectorialité-Respiratoire-Son (AIRS) Network, the 10.13039/501100004344Stichting Astma Bestrijding (SAB), the Leiden University Fund (LUF), the 10.13039/501100000691Academy of Medical Sciences (AcMedSci) and the Ghent University Special Research Fund (UGent- BOF23/DOC/013).

## Financial/Nonfinancial Disclosures

The authors have reported to *CHEST* the following: Outside this work, S. Ri. has received non-financial support from AstraZeneca, GlaxoSmithKline, and Sanofi Regeneron; S. Ra. has received salary support from the National Institute for Health and Care Research (NIHR) UK and the Charlie’s Foundation for Research. S. Ra. also declares speaker fees from GlaxoSmithKline and AstraZeneca, and conference travel support from AstraZeneca. M. E. W. has received consulting, advisory, or speaking honoraria from Allakos, Amgen, Areteia Therapeutics, Arrowhead Pharmaceutical, AstraZeneca, Avalo Therapeutics, Celldex, Connect Biopharma, Eli Lilly, Equillium, GlaxoSmithKline, Incyte, Kinaset, Kymera, Merck, MyBiometry, Pharming, Phylaxis, Pulmatrix, Rapt Therapeutics, Recludix Pharma, Regeneron, Roche/Genentech, Sanofi/Genzyme, Sentien, Sound Biologics, Tetherex Pharmaceuticals, Uniquity Bio, Upstream Bio, Verona Pharma, and Zurabio. J. C. has received grants or contracts from Regeneron, Sanofi, and Novartis. He also has received consulting fees from AstraZeneca, Amgen, Regeneron, and Sanofi and payment of honoraria from AstraZeneca, Amgen, Regeneron, and Sanofi. S. E. D. has received consultancy fees from AstraZeneca. C. E. B. has received grants and consultancy fees from 4D Pharma, Areteia, AstraZeneca, Chiesi, Genentech, GlaxoSmithKline, Mologic, Novartis, Regeneron Pharmaceuticals, Roche, and Sanofi. M. C. has received grants or contracts from American Lung Association, AstraZeneca, Gala Therapeutics, Genentech, GlaxoSmithKline, National Institutes of Health, Nocion, Novartis, Patient-Centered Outcomes Research Institute, Pulmatrix, Sanofi-Aventis, Shionogi, and Theravance Biopharma. He has also received consulting fees from Allakos, Amgen, Apogee, Apreo Health, Arrowhead Pharmaceuticals, Blueprint Medicines, Connect BioPharma, Evommune, Genentech, GlaxoSmithKline, Jasper, Kinaset, Merck, Novartis, OM Pharma, Pfizer, Pioneering Medicines, Sanofi-Aventis, Teva, Third Rock Ventures, Upstream Bio, and Verona Pharmaceuticals; honoraria from Amgen, AstraZeneca, Med Learning Group, Regeneron Pharmaceuticals, and Sanofi; and stock options from Aer Therapeutics. N. A. H. has received honoraria for serving as a consultant or advisor to GlaxoSmithKline, AstraZeneca, Genentech, Sanofi, Regeneron, Verona, and Amgen; and research grant support from GlaxoSmithKline, AstraZeneca, Genentech, Regeneron, and Sanofi. D. J. J. has received advisory board and speakers fees from AstraZeneca, Boehringer Ingelheim, Novartis, Teva, GlaxoSmithKline, Sanofi-Regeneron, and Chiesi. A. L. is an employee for AstraZeneca. N. M. is an employee and shareholder with AstraZeneca. M. E. H. is a Sanofi employee. C. T. J. H. is a former employee of Genentech. S. A. is a Novartis employee. T. S. C. H. was supported by a Wellcome Trust Fellowship (211050/Z/18/z); he reports grants from the Guardians of the Beit Fellowship, Pfizer, NIHR Oxford Biomedical Research Centre (BRC), University of Oxford, Kymab, Arcturis, and Asthma+Lung UK; and personal fees from AstraZeneca Pieris. R. W. B. has received institutional research funding from AstraZeneca, Teva, Health Research Council, Cure Kidz, and Perpetual Guardian; personal fees from AstraZeneca, Avillion, and Teva; and is Chair of the Asthma Foundation of New Zealand adolescent and adult asthma guidelines, a reviewer for GINA, and a former member of the Global Initiative for Chronic Obstructive Lung Disease board. J. K. S. has received nonrestricted research grants from AstraZeneca, European Respiratory Society Severe Heterogeneous Asthma Research collaboration—Patient Centred Clinical Research Collaboration, Register of Adult Patients with Severe Asthma for Optimal Disease Management Foundation, and ZonMw. E. W. S. has received consultancy fees from GlaxoSmithKline. I. D. P. has received honoraria for speaking at sponsored meetings from AstraZeneca, Circassia, AmgenNovartis, Chiesi, Sanofi-Regeneron, Menarini, and GlaxoSmithKline; and payments for organising educational events from AstraZeneca, GlaxoSmithKline, Sanofi-Regeneron, and Teva. He has received honoraria for attending advisory panels with Genentech, SanofiRegeneron, AstraZeneca, GlaxoSmithKline, Novartis, Teva, Merck, Circassia, and Amgen. He has received sponsorship to attend international scientific meetings from GlaxoSmithKline, AstraZeneca, Sanofi, and Regeneron. G. B. has received speaker honoraria from AstraZeneca, Boehringer Ingelheim, Chiesi, GlaxoSmithKline, Merck Sharp & Dohme, Novartis, and Sanofi-Regeneron; he is past President of the Belgian Respiratory Society. S. C. reports the following: he has received non-restricted research grants from the NIHR Oxford BRC, the Quebec Respiratory Health Research Network, the Association Pulmonaire du Québec, the Academy of Medical Sciences, AstraZeneca, bioMérieux, Circassia Niox Group, and Sanofi-Genyme-Regeneron; he is the holder of the Association Pulmonaire du Québec’s Research Chair in Respiratory medicine and is a clinical research scholar of the Fonds de recherche du Québec; he received speaker honoraria from AstraZeneca, GlaxoSmithKline, Sanofi-Regeneron, Circassia Niox Group and Valeo Pharma; he received consultancy fees for FirstThought, Apogee Therapeutics, Upstream Bio, AstraZeneca, GlaxoSmithKline, Sanofi-Regeneron, Access Biotechnology and Access Industries; he has received sponsorship to attend/speak at international scientific meetings by/for AstraZeneca and Sanofi-Regeneron. He is an advisory board member and detains stock options for Biometry Inc—a company developing a Feno device (myBiometry); he is co-inventor for the patent filed as “Method for alleviating dyspnea with neuromodulation.” He advised the Institut national d'excellence en santé et services sociaux (INESSS) for an update of the asthma general practice information booklet for general practitioners as well as therapeutic indications for Enerzair, and is a member of the asthma steering committee of the Canadian Thoracic Society. None declared (F. L. M., S. M.-L., D. C., A. M.).

## Supplementary Data

e-Online Data
