# Supplementary material for: Impact of Sex on Prediction of Asthma Attacks by Clinical Risk Factors and Type 2 Biomarkers
Source: Chest. 2026 Jan 31;169(6):1464–75. doi: 10.1016/j.chest.2025.12.049 (PMC13269683; doi:10.1016/j.chest.2025.12.049)
Supplement: e-Online Data [file mmc1.docx]

**SUPPLEMENTARY MATERIAL**

**e-Table 1: Additional study-level information for the trials that provided individual participant data***

| **Trial(s) reported** [registration no.] | **Study design** | **Key inclusion criteria** | **Reversibility inclusion criteria** | **Recruitment biomarker targets** | **Investigator blinding to biomarkers** | **Endpoints** |
| --- | --- | --- | --- | --- | --- | --- |
| **AZISAST (AZIthromycin in Severe ASThma)**  [[NCT00760838](https://classic.clinicaltrials.gov/ct2/show/NCT00760838)] | Randomised,  placebo-controlled double-blind  parallel-group | • Age 18–75  • GINA step 4 or 5  • High doses of ICS/LABA  • At least 2 severe asthma exacerbations or LRTI requiring antibiotics within the previous 12 months | Historical, 1 of:  i) postBD reversibility FEV1;  ii) BPT test positive;  iii) PEF variability | All patients: FeNO level below the upper limit of normal according to gender, atopic status, and smoking history^13^ | - | Primary  • Severe asthma exacerbations and/or LRTI requiring antibiotics  Secondary  • FEV1, PEF, AQLQ and ACQ |
| **Benralizumab  phase 2b** [[NCT01238861](https://clinicaltrials.gov/study/NCT01238861)] | Randomised, placebo-controlled, double-blind,  parallel-group dose-ranging | • Aged 18–75  • Medium-dose high-dose ICS/LABA for at least 1 year • History of two to six severe exacerbations in the past year  • FEV1 40%-90%, reversibility • ACQ-6 score of 1.5 or higher | Past 36 months, 1 of:  i) postBD reversibility, FEV1 or FVC, 200 mL and 12%;  ii) BPT test positive; | - | Patients, site personnel, and sponsor  blinded to eosinophil counts post-randomization | Primary • Severe asthma annual exacerbation rate in eosinophilic individuals  Secondary • Change from baseline in FEV1, ACQ-6, overall symptom score, and AQLQ |
| **CAPTAIN (Clinical Study in Asthma Patients Receiving Triple Therapy in a Single Inhaler)** [[NCT02924688](https://clinicaltrials.gov/study/NCT02924688)] | Randomised, double-blind, parallel-group, phase 3A | • Aged ≥18 years  • ACQ-6 ≥1.5 • ICS/LABA (>250 μg per day fluticasone or equivalent) for at least 12 consecutive weeks  • Documented health-care contact or temporary change in asthma therapy for acute asthma within 1 year  • FEV1 30%-85%, reversibility | Baseline, all patients:  postBD reversibility, FEV1, 200 mL and 12%; | **-** | - | Primary  • Change from baseline in FEV1  Secondary  • Annualised rate of moderate and/or severe asthma exacerbations |
| **COSTA** [[NCT01582503](https://clinicaltrials.gov/study/NCT01582503)] | Randomised, placebo-controlled double-blind, parallel-group, phase 2 | • Aged 18–75 years  • FEV1 40–80 %, reversibility • Daily use of ICS (≥400 μg/day total daily dose of FP) and second controller for 3 months  • ACQ-5 ≥1.5  • History of at least one severe asthma exacerbation in the 18 months prior | Past 24 months, 1 of:  i) postBD reversibility, FEV1 or FVC, 12%;  ii) BPT test positive; | All patients: At least one positive aero-allergen or a total serum IgE ≥75 IU/mL | - | Primary outcome  • Annualised rate of severe asthma exacerbations  Secondary outcomes  • FEV1 and the change in asthma symptoms |
| **…Trial** | **Design** | **Key inclusion criteria** | **Reversibility** | **Biomarker target** | **Biom blinding** | **Endpoints** |
| **DREAM (Dose Ranging Efficacy And safety with Mepolizumab)** [[NCT01000506](https://clinicaltrials.gov/study/NCT01000506)] | Randomised,  placebo-controlled, double-blind, parallel-group,  dose-ranging | • Age 12–74 years  • History of two or more severe exacerbations  • FEV1 <80% • At least 880 μg fluticasone propionate/day, with or without OCS. | Past 12 months, 1 of:  i) postBD reversibility FEV1; 12% and 200 mL ii) BPT test positive;  iii) FEV1 inter-visit variability, 20%  iii) PEF diurnal variability, 20% | All patients, past 12 months, 1 of:  i) sputum eosinophil count ≥3%,  ii) FENO ≥50 ppb,  iii) blood eosinophil ≥0·3×10⁹/L. | Patients, site personnel, and sponsor  blinded to eosinophil counts post-randomization | Primary outcome  • Rate of severe clinically significant asthma exacerbations  Secondary outcomes  • Rate of exacerbations, blood and sputum eosinophil counts, FEV1, AQLQ and ACQ |
| **Dupilumab phase 2b** [[NCT01854047](https://clinicaltrials.gov/study/NCT01854047)] | Randomised, placebo-controlled, double-blind, parallel-group, dose-ranging | • Adults (aged ≥18 years)  • Treatment with medium-to-high- dose ICS/LABA at least 1 month  • FEV1 of 40–80%  • ACQ-5 ≥ 1·5 • At least one severe asthma exacerbation | Baseline, all patients:  postBD reversibility, FEV1, 200 mL and 12%; | **-** | - | Primary endpoint  • Change in FEV1 in patients with baseline blood eosinophil ≥300  Secondary endpoints  • Change from baseline in FEV1; annualised severe asthma exacerbation rate; ACQ-5, AQLQ, FeNO |
| **EXTRA** [[NCT00314574](https://clinicaltrials.gov/study/NCT00314574)] | Randomised, placebo-controlled double-blind, parallel-group, | • Aged 12 to 75 years  • History of severe allergic asthma for at least 1 year  • FEV1 of 40-80% • Asthma was not well-controlled despite treatment with high-dose ICS/LABA with or without other controllers (including OCS), based on NAEPP guidelines. | No reversibility criteria: “Physician-diagnosed asthma” | All patients, past 12 months:  At least one positive aero-allergen or a total serum IgE 30-<700 IU/mL | - | Primary end point  • Rate of severe asthma exacerbations  Secondary end points  • Change in total asthma symptom severity score (TASS), change in mean puffs per day of albuterol; and change AQLQ |
| **LAVOLTA I, II** [[NCT01867125](https://clinicaltrials.gov/study/NCT01867125), [NCT01868061](https://clinicaltrials.gov/study/NCT01868061)] | Randomised,  placebo-controlled, double-blind,  parallel-group, replicate, phase 3, | • Aged 18–75 years  • FEV1 40–80% • ICS (500–2000 μg per day fluticasone propionate or equivalent) for at least 6 months and at least one additional controller medication.  • ACQ-5 ≥1·5 | Baseline, all patients:  postBD reversibility, FEV1, 12%; | **-** | Patients, site personnel, and sponsor  blinded to eosinophil counts, FeNO, and periostin post-randomization | Primary endpoint  • Rate of severe asthma exacerbations in biomarker-high  Secondary endpoints  • Absolute changeFEV1; time to first asthma exacerbation; AQLQ, ACQ-5 |

| **…Trial** | **Design** | **Key inclusion criteria** | **Reversibility** | **Biomarker target** | **Biom blinding** | **Endpoints** |
| --- | --- | --- | --- | --- | --- | --- |
| **LUSTER I, II** [[NCT02555683](https://clinicaltrials.gov/study/NCT02555683), [NCT02563067](https://clinicaltrials.gov/study/NCT02563067)] | Randomised, placebo-controlled, double-blind, parallel-group, replicate, phase 3 | • Aged 12 years or older  • Uncontrolled asthma on dual/triple asthma therapy with medium/high dose ICS with up to two controllers with or without OCS  • History of two or more asthma exacerbations within the previous 12 months. • FEV1 ≤80% | Past 24 months, 1 of:  i) postBD reversibility, FEV1 or FVC, 200 mL and 12%;  ii) BPT test positive; | • 2/3 : blood eosinophil count ≥0·25×10⁹/L  • 1/3 : blood eosinophil count <0·25×10⁹/L. | - | Primary endpoint  • Number of moderate to severe asthma exacerbations per patient year both in patients with high blood eosinophil counts  (≥250 cells/μL) and in all patients  Secondary endpoints  • Change from baseline in FEV1, ACQ-5, AQLQ |
| **LUTE, VERSE** [[NCT01545440](https://clinicaltrials.gov/study/NCT01545440), [NCT01545453](https://clinicaltrials.gov/study/NCT01545453)] | Randomised, placebo-controlled double-blind, replicate studies, phase IIb. Amended protocol and early termination | • Patients aged 18–75 years  • Daily use of 500–2000 μg/day of fluticasone and a second asthma controller medication ≥12 months • FEV1 40–80% • ACQ-5 ≥1.5 | Baseline, all patients:  postBD reversibility, FEV1, 12%; | **-** | Patients, physicians and site staff were blinded to FeNO and periostin | Primary endpoint  • Rate of severe asthma exacerbations  Secondary endpoints  • Change in FEV1 from baseline, time to first exacerbation,  AQLQ |
| **MILLY** [[NCT00930163](https://clinicaltrials.gov/study/NCT00930163)] | Randomised, placebo-controlled double-blind, parallel-group | • Age 18-65 years  • For at least 6 months of ICS (≥200 and ≤1000 μg of inhaled fluticasone propionate daily) • ACQ5 ≥1.5  • FEV1 40%-80%, | Baseline, all patients:  postBD reversibility, FEV1, 12%; | **-** | - | Primary outcome  • Relative change in FEV1  Secondary outcomes  • Rates of moderate and severe exacerbations, PEF, ACQ5, ACDD, and use of rescue medication |
| **NAVIGATOR** [[NCT03347279](https://clinicaltrials.gov/study/NCT03347279)] | Randomised, placebo-controlled double-blind parallel-group,  phase 3 | • Age 12-80 years  • Medium/high-dose ICS for at least 12 months and at least one additional controller, with or without oral glucocorticoids, for at least 3 months before  • FEV1 <80% • At least two exacerbations in the 12 months before | Baseline, all patients:  postBD reversibility, FEV1, 200 mL and 12%; | • 50% : blood eosinophil count ≥0·3×10⁹/L  • 50% : blood eosinophil count <0·3×10⁹/L. | Patients, physicians and site staff were blinded to FeNO | Primary endpoint  • Rate of severe asthma exacerbations  Secondary endpoints  • Change in FEV1 from baseline, time to first exacerbation,  AQLQ |

| **…Trial** | **Design** | **Key inclusion criteria** | **Reversibility** | **Biomarker target** | **Biom blinding** | **Endpoints** |
| --- | --- | --- | --- | --- | --- | --- |
| **NOVEL START** [[ACTRN12615 000999538](https://www.anzctr.org.au/Trial/Registration/TrialReview.aspx?ACTRN=12615000999538)] | Randomised, open-label, controlled parallel group trial | • Age 18 to 75 years  • SABA as the sole asthma therapy in the previous 3 months on at least two occasions, but on an average of two or fewer occasions per day in the previous 4 weeks | No reversibility criteria: “Self-reported asthma” | **-** | - | Primary outcome  • Annualised rate of asthma severe/moderate exacerbations Secondary outcomes • Number of exacerbations, number of severe exacerbations, ACQ-5, FeNO, oral prednisone use |
| **PACT** [[NCT00272506](https://clinicaltrials.gov/study/NCT00272506)] | Randomised,  placebo-controlled, double-blind, parallel group | • Age 6-14 years  • FEV1 ≥70% predicted • Mild-moderate persistent asthma | No reversibility criteria: all patients required positive BPT at visit 2 | **-** | - | Primary outcome  • Percent of asthma control days  Secondary analyses • Percent of episode-free days, number of severe exacerbations and time to the first exacerbation, ACQ |
| **PATHWAY** [[NCT02054130](https://clinicaltrials.gov/study/NCT02054130)] | Randomised, placebo-controlled double-blind,  parallel group | • Age 18 to 75  • Asthma not well controlled despite treatment with medium/high dose LABA/ICS  at least 6 months  • At least two severe asthma exacerbations  • FEV1 40%-80% • ACQ-6 ≥1.5 | Past 12 months, all patients:  postBD reversibility, FEV1, 200 mL and 12%; | • ≥50% : blood eosinophil count | - | Primary endpoint  • Annualised rate of severe asthma exacerbations Secondary endpoints  • Changes FEV1, ACQ-6, AQLQ, asthma symptom score, and FVC, annualized rate of exacerbations, time to the first exacerbation |

| **…Trial** | **Design** | **Key inclusion criteria** | **Reversibility** | **Biomarker target** | **Biom blinding** | **Endpoints** |
| --- | --- | --- | --- | --- | --- | --- |
| **PRACTICAL** [[ACTRN12616 000377437](https://www.anzctr.org.au/Trial/Registration/TrialReview.aspx?ACTRN=12616000377437)] | Randomised, open-label, controlled  parallel-group, pragmatic trial | • Aged 18 to 75  • SABA reliever alone in the past 12 weeks, symptoms or need for SABA on ≥2 occasions in the past 4 weeks, or a history of a severe asthma exacerbation in the past 52 weeks • SABA reliever with low/moderate doses of ICS in the past 12 weeks, partly or well controlled or uncontrolled asthma with poor adherence | No reversibility criteria: “Self-reported asthma” | **-** | - | Primary outcome  • Number of severe asthma exacerbations per patient per year Secondary outcomes  • Proportion of patients with a least one severe exacerbation; time to first exacerbation; ACQ-5, FEV1, FeNO |
| **QUEST** [[NCT02414854](https://clinicaltrials.gov/study/NCT02414854)] | Randomised, placebo-controlled, double-blind, parallel-group,  phase 3 | • Age 12 years or older  • Medium/high-dose ICS/LABA plus up to 2 controllers  • FEV1 80% or less, reversibility  • ACQ-5 ≥1.5 • Severe asthma exacerbation in the previous year | Baseline, all patients:  postBD reversibility, FEV1, 200 mL and 12%; | **-** | - | Primary efficacy end points  • Annualised rate of severe exacerbation events and the absolute change from baseline in the FEV1 Secondary end points  • Percentage change from baseline in the FEV1 |
| **STRATOS I, II** [[NCT02161757](https://clinicaltrials.gov/study/NCT02161757), [NCT02194699](https://clinicaltrials.gov/study/NCT02194699)] | Randomised, placebo-controlled, double-blind, parallel-group, replicate trials, phase 3 | • Aged 12–75 years  • Medium/high dose ICS/LABA for at least 3 months  • Additional controller medications if required | Baseline, all patients:  postBD reversibility, FEV1, 200 mL and 12%; | **-** | - | Primary endpoint  • Annualised severe asthma exacerbation rate  Secondary endpoints  • FEV1, ACQ-6, AQLQ,  time to first exacerbation; proportion of participants  with one or more exacerbations |

*Benralizumab phase 2a removed from the table, as did not provide individual participant data. BD, bronchodilator; BPT, bronchial provocation test; ACQ, asthma control questionnaire; AQLQ, asthma quality of life questionnaire; FEV_1_, forced expiratory volume in 1^st^ second; FVC, forced vital capacity; ICS, inhaled corticosteroid; LABA, long-acting beta-agonist; LAMA, long-acting muscarinic antagonist; PEF, peak expiratory flow.

**e-Table 2: overview of individual items in the ACQ-5 questionnaire by sex**

|  | Females | Males | p-value |
| --- | --- | --- | --- |
| Sleep awakenings  *On average, during the past week, how often were you woken by your asthma during the night?* | 1.92 | 1.64 | **<0.001** |
| Morning symptoms  *On average, during the past week, how bad were your asthma symptoms when you woke up in the morning?* | 2.35 | 2.18 | **<0.001** |
| Activity limitation  *In general, during the past week, how limited were you in your activities because of your asthma?* | 2.37 | 2.17 | **<0.001** |
| Dyspnea  *In general, during the past week, how much shortness of breath did you experience because of your asthma?* | 2.68 | 2.51 | **<0.001** |
| Wheezing  *In general, during the past week, how much of the time did you wheeze?* | 2.44 | 2.31 | **0.01** |

**e-Table 3: Interaction analysis of sex with baseline clinical characteristics and biomarkers.** For each individual trait, both the interaction term and corresponding *P*-value (False Discovery Rate-analysis) are displayed

|  | Age | BMI | Smoking | Atopy | Airborne allergen | CRSsNP | CRSwNP | Treatment step | ACQ | Attack history | FEV1% | FEV1 reversibility | Eosinophils | FeNO | IgE |
| --- | --- | --- | --- | --- | --- | --- | --- | --- | --- | --- | --- | --- | --- | --- | --- |
| *Interaction*  *term* | **1.06** | **1.00** | **1.00** | **1.17** | **0.82** | **1.34** | **1.27** | **0.51** | **0.94** | **1.76** | **1.00** | **1.01** | **1.05** | **1.29** | **1.03** |
| *p-value* | 0.56 | 0.98 | 0.98 | 0.60 | 0.50 | 0.42 | 0.46 | 0.18 | 0.93 | **0.03** | 0.98 | 0.97 | 0.93 | 0.42 | 0.93 |

**
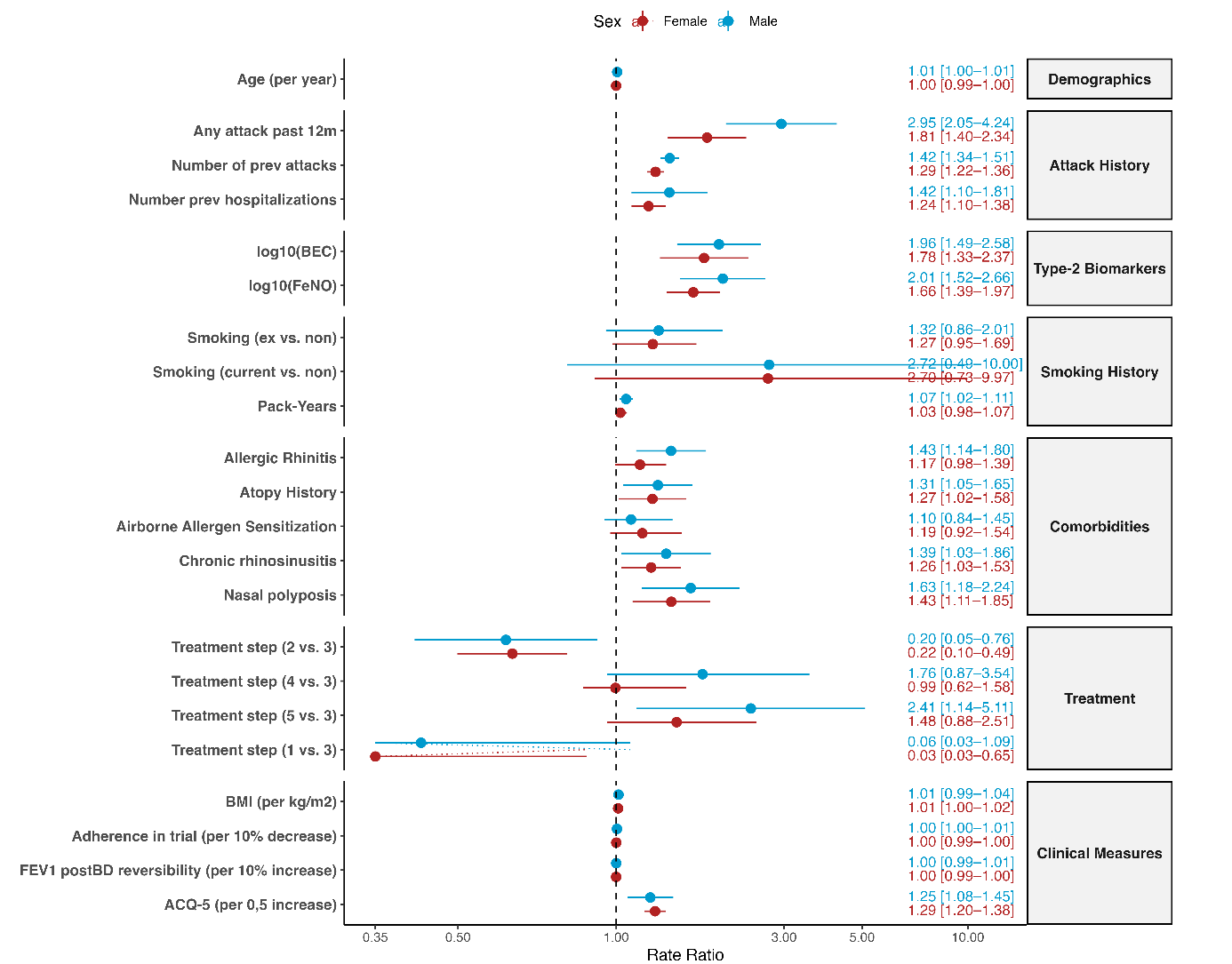
**

**e-Figure 1: Rate ratios (95% CI) of Annualized Severe Asthma Attack Rate (ASAAR) from univariable models according to different clinical variables and type-2 biomarkers.** Univariable models were computed correcting for enrolled trial and duration of follow-up as offset variable.

**
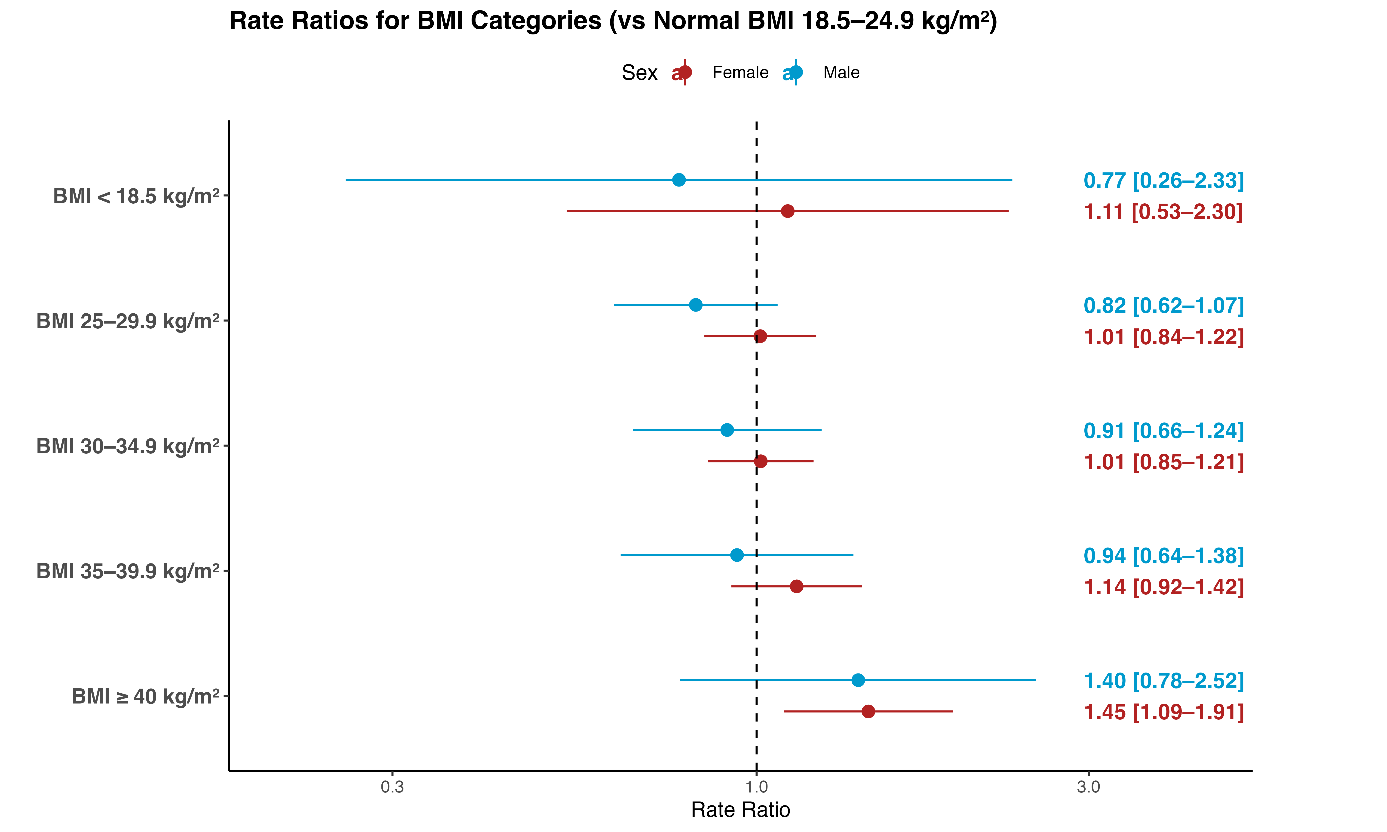
e-Figure 2: Rate ratios (95% CI) of the ASAAR according to category of BMI, relative to normal weight (BMI 18.5-25 kg/m²).** BMI categories are stratified according to WHO classification. Rate ratios are derived from multivariable negative binomial models, adjusted for asthma attack in the past year (yes vs no), asthma severity (treatment step 1–5), FEV1 prebronchodilator, ACQ-5 symptom score, BEC, FeNO, adjusted enrolled trial as a factor, and follow-up duration as an offset variable. ACQ-5=5-item Asthma Control Questionnaire. BEC= blood eosinophil count, FeNO=fractional exhaled nitric oxide


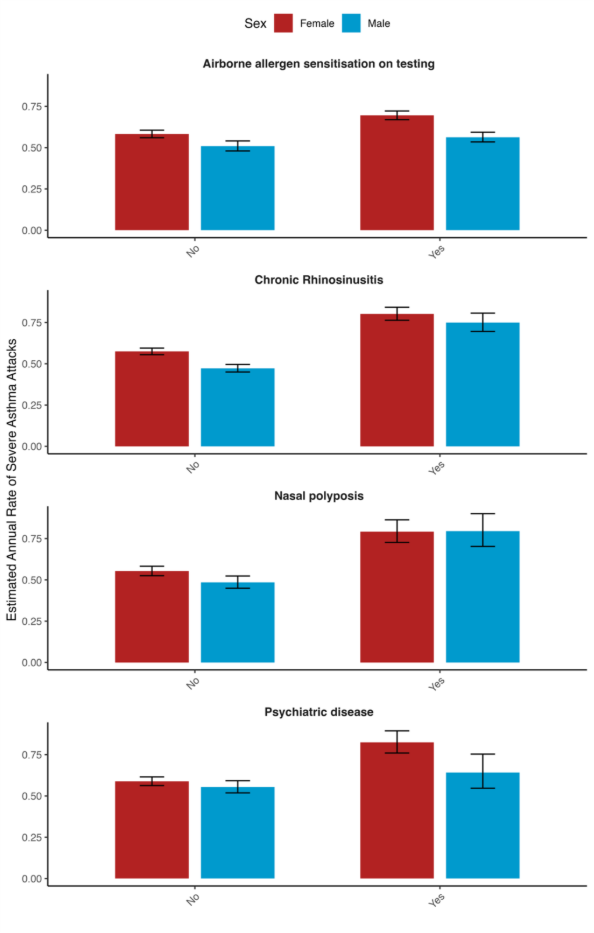


**e-Figure 3: Sex-stratified Annualized Severe Asthma Attack Rate (ASAAR) by airborne allergen sensitisation (A), chronic rhinosinusitis (B), nasal polyposis (C) and psychiatric disease (D).** Estimates use following reference values: mean age, mean BMI, mean baseline ACQ-5 score, mean FEV1 prebronchodilator, mean FeNO, mean BEC, GINA treatment step 4, CAPTAIN trial and duration of follow-up. ACQ-5=5-item Asthma Control Questionnaire. BEC= blood eosinophil count, FeNO=fractional exhaled nitric oxide


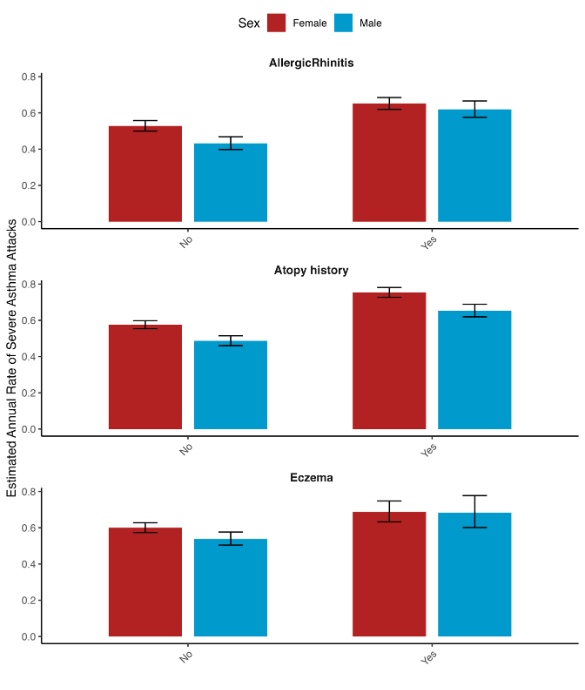


**e-Figure 4: Sex-stratified Annualized Severe Asthma Attack Rate (ASAAR) by allergic rhinitis (A), atopy history (B) and eczema (C).** Estimates use following reference values: mean age, mean BMI, mean baseline ACQ-5 score, mean FEV_1_ prebronchodilator, mean FeNO, mean BEC, GINA treatment step 4, CAPTAIN trial and duration of follow-up. ACQ-5=5-item Asthma Control Questionnaire. BEC= blood eosinophil count, FeNO=fractional exhaled nitric oxide


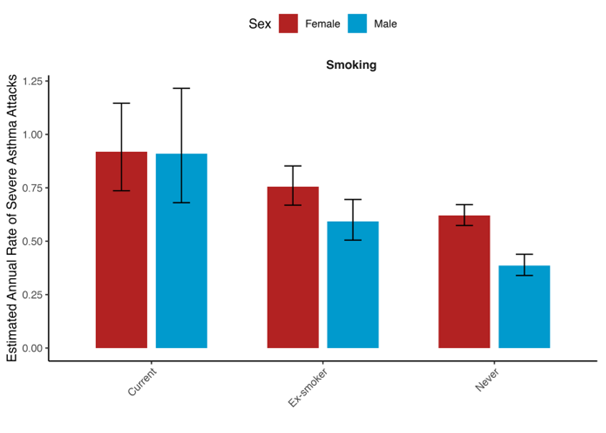


**e-Figure 5: Sex-stratified Annualized Severe Asthma Attack Rate (ASAAR) by smoking status.** Estimates use following reference values: mean age, mean BMI, mean baseline ACQ-5 score, mean FEV_1_ prebronchodilator, mean FeNO, mean BEC, GINA treatment step 4, CAPTAIN trial and duration of follow-up. ACQ-5=5-item Asthma Control Questionnaire. BEC= blood eosinophil count, FeNO=fractional exhaled nitric oxide


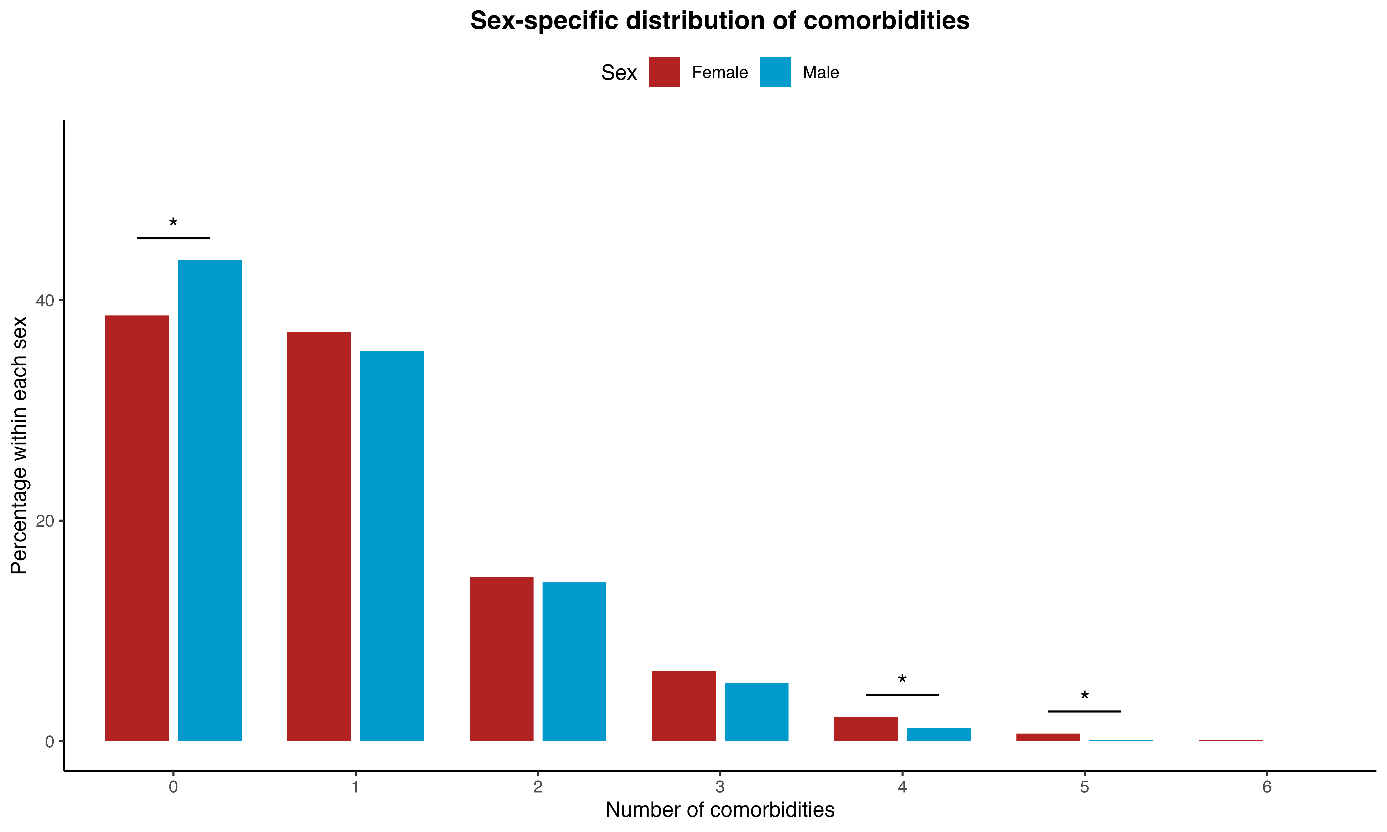


**e-Figure 6:** **Distribution of the number of comorbidities by sex.** Comorbidities included in analysis were allergic rhinitis, chronic rhinosinusitis, nasal polyps, eczema, psychiatric disease and obesity (BMI >30kg/m^2^)


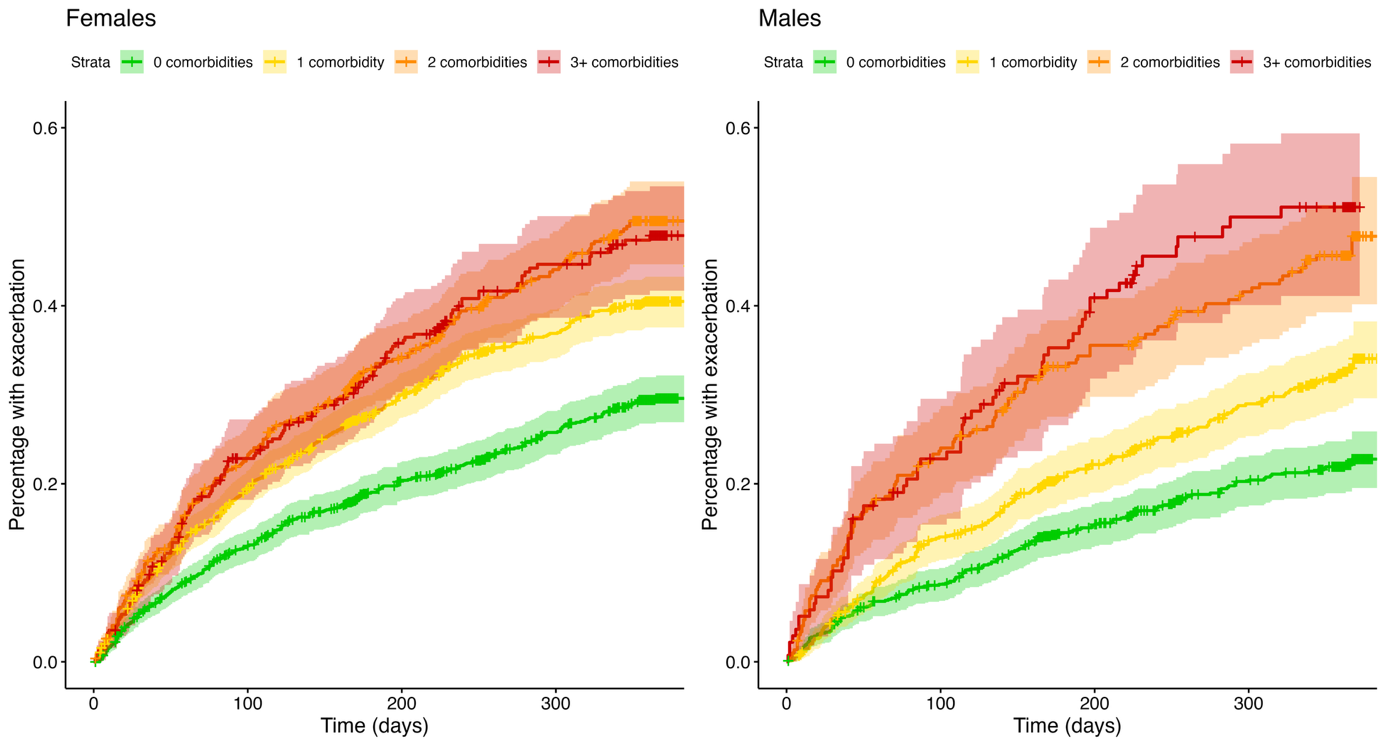


**e-Figure 7:** **Proportion of male and female asthma patients with severe asthma attack(s) in ORACLE2 over one year of prospective follow-up by number of comorbidities.** Comorbidities included in analysis were allergic rhinitis, chronic rhinosinusitis, nasal polyps, eczema, psychiatric disease and obesity (BMI >30kg/m^2^). Trials with <12 months follow-up (PACT, LUSTER-1, LUSTER-2) were excluded. Shaded areas indicate 95% CIs.


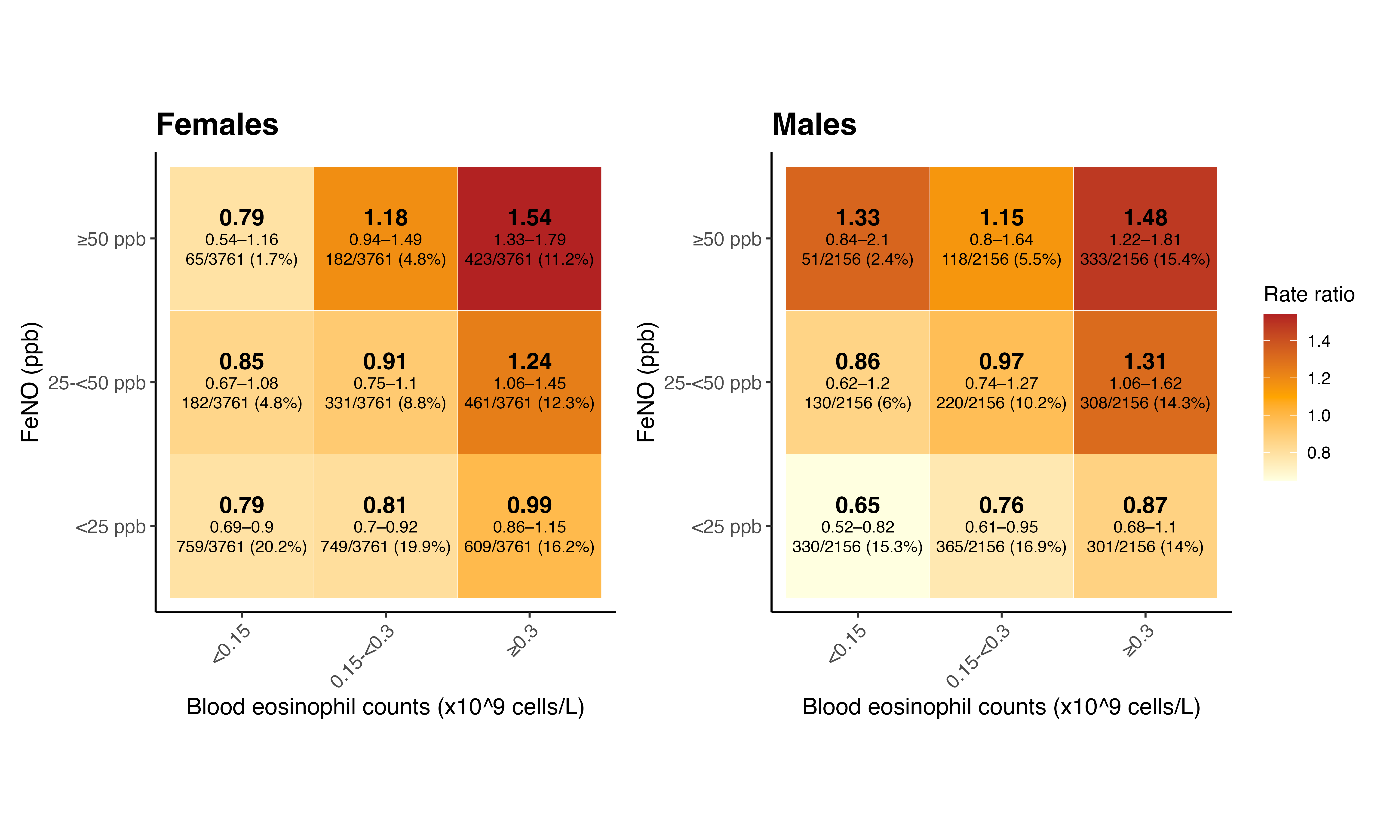


**e-Figure 8: Sex-stratified Annualized Severe Asthma Attack Rate (ASAAR) according to categories of BEC and FeNO.** FeNO was stratified to low (<25ppb), intermediate (25-50 ppb) or high (≥50 ppb). A negative binomial model was fitted for each imputed dataset for the main predictors excluding FeNO and BEC. Estimated risk was calculated using following reference values: mean age, mean BMI, mean baseline ACQ-5 score, mean FEV1 % prebronchodilator, GINA treatment step 4, CAPTAIN trial and duration of follow-up.


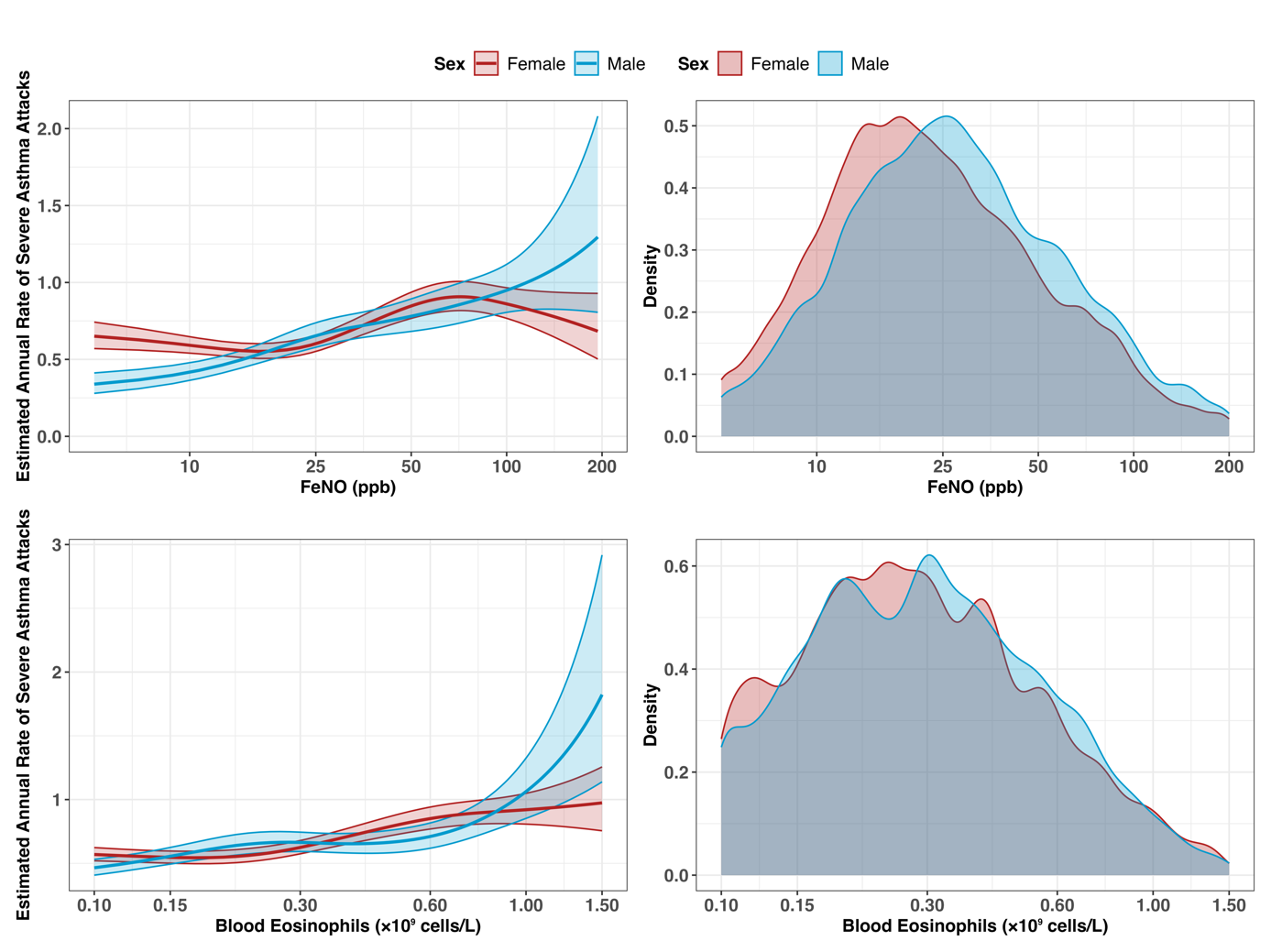


**e-Figure 9: Estimated Annualized Severe Asthma Attack Rate (ASAAR) according to FeNO (top) and BEC (bottom).** Left plots: spline plots of the relationship between FeNO (ppb) and blood eosinophil count (×10^9^ cells per L) and the estimated ASAAR. Right plots: probability density of baseline FeNO and baseline blood eosinophil count observations by sex.

**
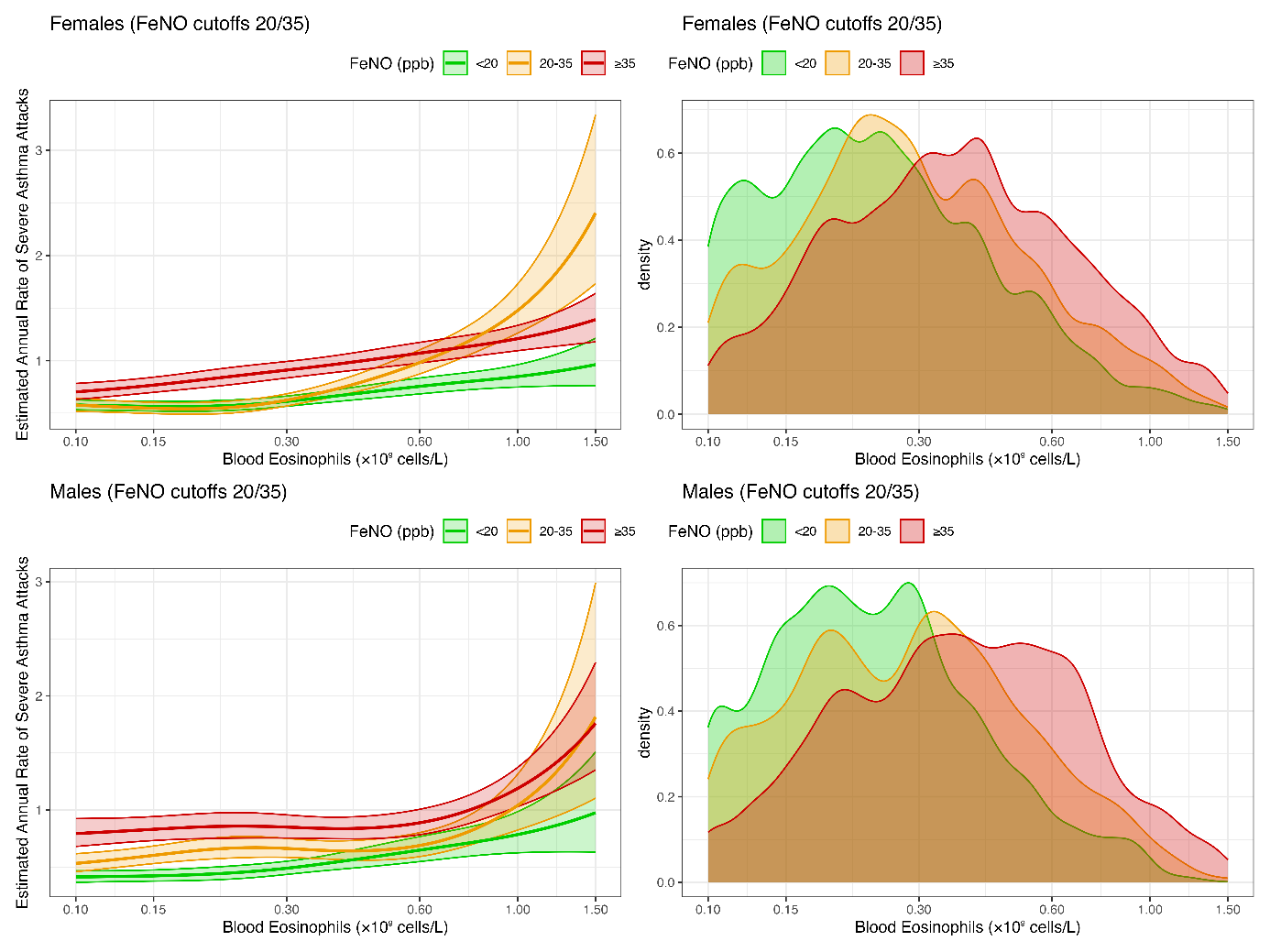
**

**e-Figure 10: Estimated Annualized Severe Asthma Attack Rate (ASAAR) according to combinations of FeNO and BEC in females (top) and males (bottom).** FeNO was stratified to low (<20ppb), intermediate (20-35 ppb) or high (≥35 ppb). Probability density of baseline blood eosinophil count observations per group of baseline FeNO value in the imputed datasets are displayed to the right of the spline curves.


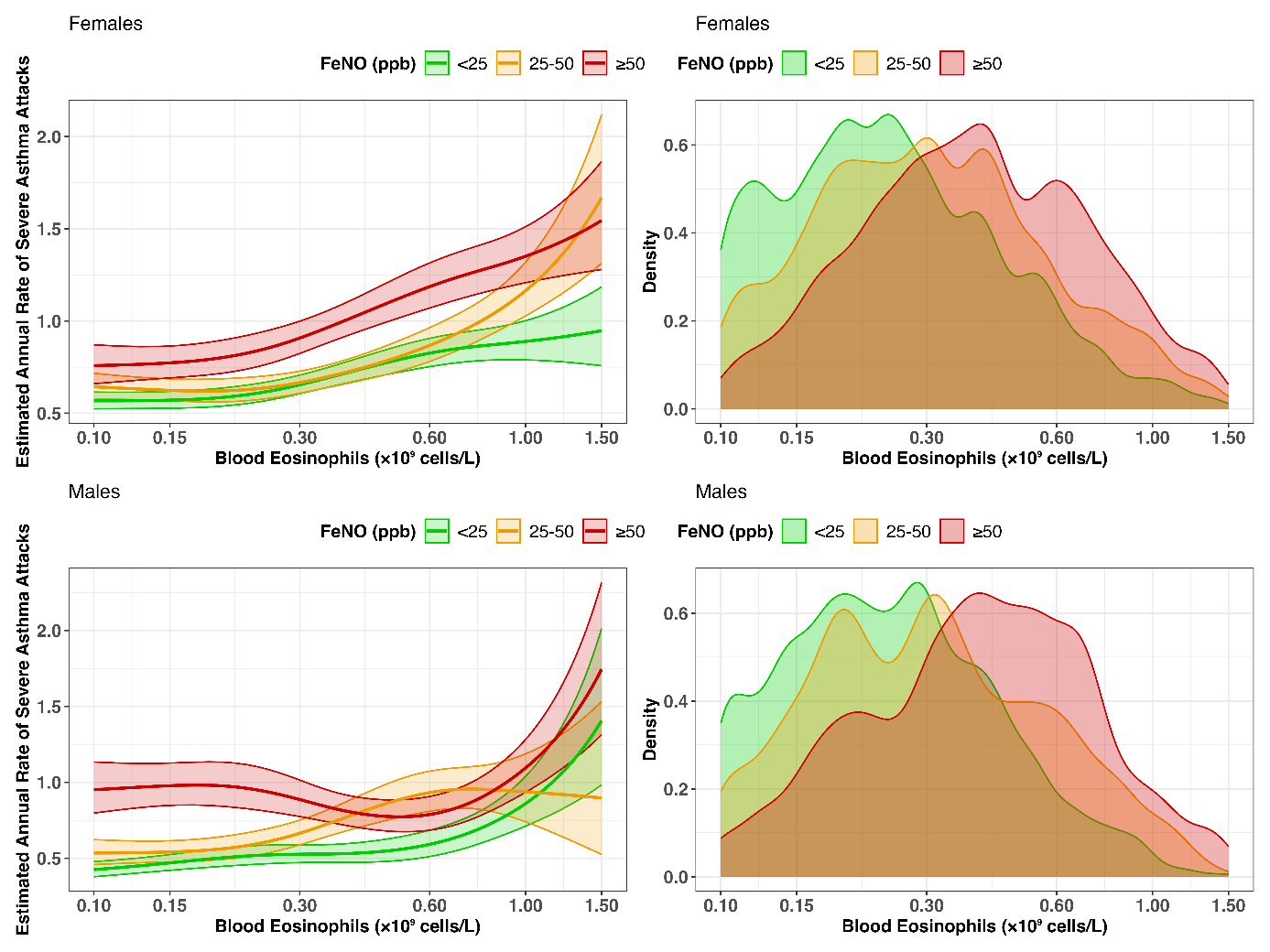
**e-Figure 11: Estimated Annualized Severe Asthma Attack Rate (ASAAR) according to combinations of FeNO and BEC in females (top) and males (bottom).** FeNO was stratified to low (<25ppb), intermediate (25-50 ppb) or high (≥50 ppb). Probability density of baseline blood eosinophil count observations per group of baseline FeNO value in the imputed datasets are displayed to the right of the spline curves.


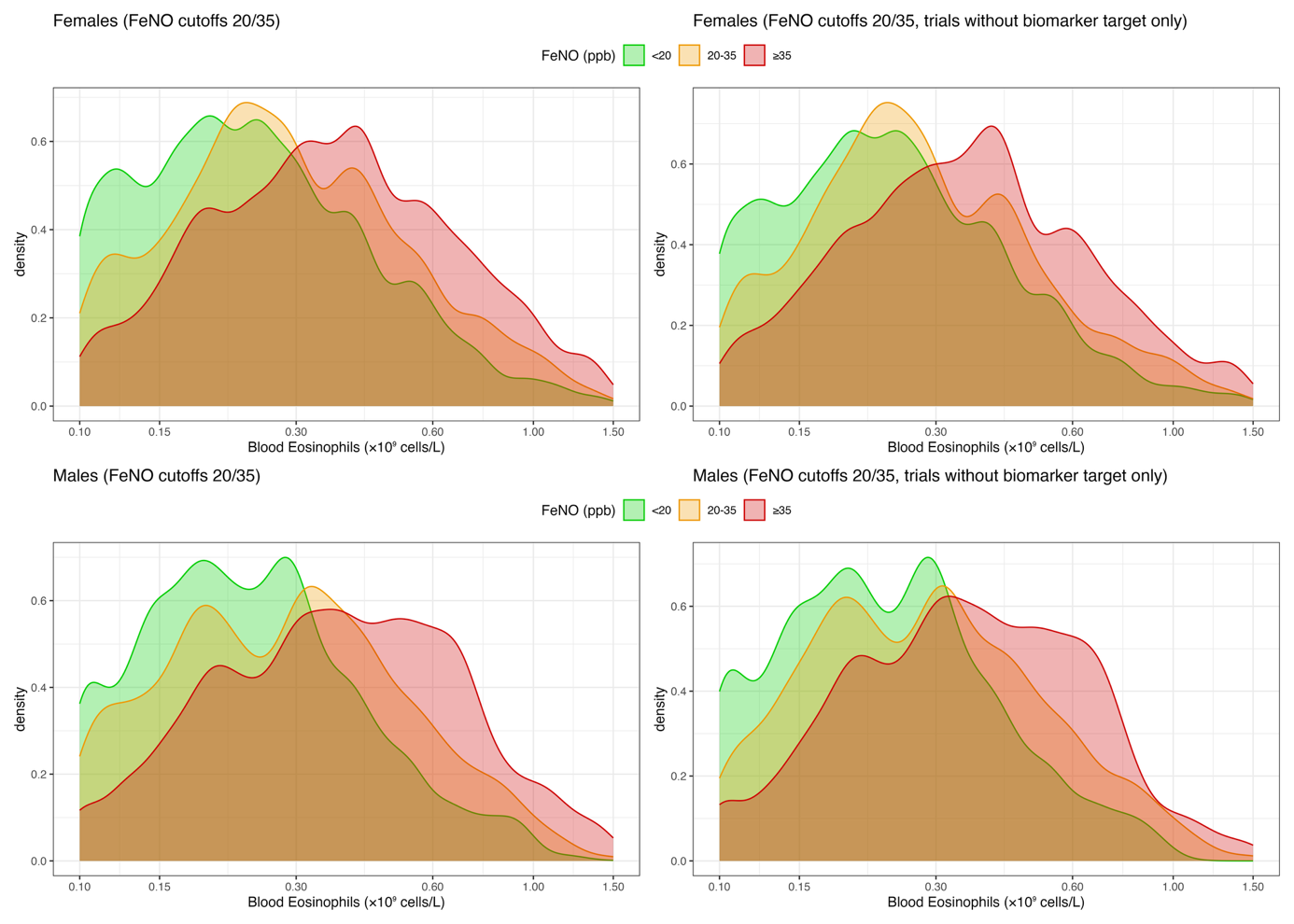
**e-Figure 12: Probability density of baseline blood eosinophil count observations per group of baseline FeNO value in the imputed datasets.** Probability density plots are displayed for the complete study groups (left) and the subgroup excluding patients in trials with a T2-biomarker target (right). The trials without T2-biomarker target were CAPTAIN, Dupilumab 2b, LAVOLTA-1, LAVOLTA-2, LUTE, MILLY, Novel START, PACT, PRACTICAL, QUEST, STRATOS-1, STRATOS-2 and VERSE.

**e-Table 4: overview of FeNO and BEC in complete study group and subgroup excluding patients in trials with a T2-biomarker target**

|  | N (Female/Male) | Females | Males |
| --- | --- | --- | --- |
| FeNO, median ppb (IQR)  ** All trials*  ** Trials without T2-biomarker target* | 4140/2370  2710/1583 | 22 (13-39)  21 (13-38) | 26 (16-47)  26 (15-46) |
| BEC, median ppb (IQR)  ** All trials*  ** Trials without T2-biomarker target* | 4140/2370  2710/1583 | 240 (140-410)  240 (140-400) | 260 (150-430)  260 (150-420) |
